# The 10^th^ Barossa meeting: Cell Signalling to Cancer Medicine

**DOI:** 10.1038/s41419-024-06614-9

**Published:** 2024-03-21

**Authors:** Winnie L. Kan, Barbara J. McClure, Christopher N. Hahn, Jason A. Powell

**Affiliations:** 1 Centre for Cancer Biology, University of South Australia and SA Pathology, Adelaide, SA 5000 Australia; 2 Adelaide Medical School, Faculty of Health Sciences, University of Adelaide, Adelaide, SA 5000 Australia

**Keywords:** Extracellular signalling molecules, Preclinical research

After a 2-year hiatus due to the COVID-19 pandemic we welcomed back the 10th Barossa meeting “Cell Signalling to Cancer Medicine” during November 2023 in the Barossa Valley, a world class wine producing area with exceptional cellar doors and local gourmet food (Fig. 1A). The meeting was co-hosted by Centre for Cancer Biology, South Australian Immunogenomics Cancer Institute and Flinders University and brought together first class international and national leaders and ECRs to share new information and perspectives on fundamental discoveries with the overarching goal to provide new therapeutic options for cancer therapy.

**Fig. 1:**
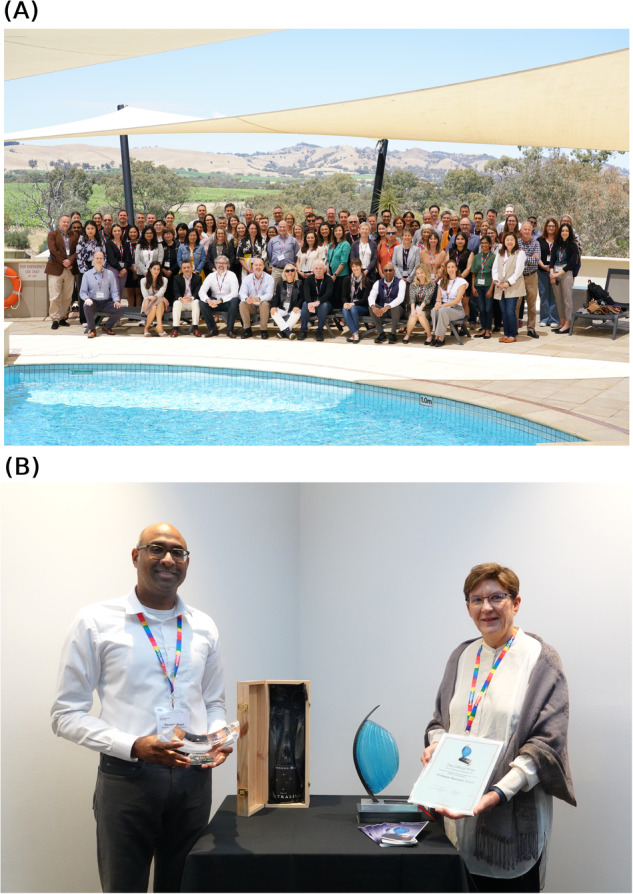
The 10th Barossa meeting. **A** Delegates of the 10^th^ Barossa meeting: Cell Signalling to Cancer Medicine. **B** The 2023 Clifford Prize for Cancer Research was awarded to Professor Ravindra Majeti. This international award recognises Professor Majeti’s international excellence in cancer research and was presented by Dr Jill Lipsett (Clinical Services Director of SA Pathology, Central Adelaide Local Health Network). Left to right; Professor Ravindra Majeti and Dr Jill Lipsett.

## Cell reprogramming in cancer

Ravindra Majeti (Stanford University, USA) opened the meeting with a stimulating talk on a new cancer vaccination approach based on direct myeloid-lineage reprogramming of haematological and solid tumour cells into tumour-reprogramming antigen presenting cells, which present endogenous tumour-associated antigens to stimulate T-cells for tumour eradication. Importantly, TR-APCs generated from primary B-cell acute lymphoblastic leukemia (B-ALL) patient samples can activate autologous T-cells, demonstrating the clinical feasibility of this reprogramming approach. Utilising zebrafish models, Andrew Cox (Peter MacCallum Cancer Institute, Australia), described the role of the transcription factor nuclear factor erythroid 2-related factor 2/Kelch-like ECH-associated protein 1 pathway in regulating lysosomal biogenesis and hepatocyte plasticity in liver cancer.

## Cell signalling networks

John Scott (University of Washington Medical Center, USA) highlighted the importance of dysregulated spatial signalling in driving adolescent liver cancer fibrolamellar carcinoma. Using proximity proteomics and live-cell imaging they demonstrate that the oncogenic fusion between the chaperonin binding domain of Hsp40 (DNAJ) with the catalytic core of protein kinase A (PKAc) is not constrained by A-kinase anchoring proteins and thus phosphorylates a unique array of substrates. David Croucher (Garvan Institute, Australia) utilised mathematical modelling and longitudinal single-cell imaging to demonstrate that a chemoresistant population of cancer cells can emerge solely through the inherently noisy process of gene expression, which is amplified by the non-linear behaviour of apoptotic signalling. Antonella Papa (Monash University, Australia) described an elegant mouse model where knock-in of PTEN and PI3K mutants drive an aggressive breast cancer which exhibits reduced overall survival compared to the single knock-ins. Amelia Parker (Garvan Institute, Australia) generated a spatial proteomic and transcriptomic map of squamous cell carcinoma of the lung to demonstrate that extracellular matrix (ECM) remodelling programs occur early in disease to activate intracellular signalling in tumour and stromal cells to drive further ECM remodelling and tumour progression. Using single cell approaches Teresa Sadras (Peter MacCallum Cancer Institute, Australia) demonstrated that in cytokine receptor-like factor 2-driven B-ALL, JAK2/STAT and RAS/ERK activating lesions are mutually exclusive, comprising distinct and competing subclones with different drug dependencies.

## Cancer immunology and signalling

Laura Mackay (Peter Doherty Institute, Australia) described identification of a distinct gene signature for tissue resident memory T-cells (TRM) that associates with improved prognosis in breast cancer, and demonstrated modulation of candidate master regulators have potential to reprogram dysfunctional exhausted T-cells, increase TRM and reduce clinical disease. Sean Porazinski (Garvan Institute, Australia) described how anti-fungal Itraconazole combined with chemotherapy hinders metastasis in a murine intrasplenic pancreatic cancer model.

## Spatial profiling of cancer

Jasmine Plummer (St Jude’s Children’s Research Hospital, USA) explained how US-born Black and immigrant Black populations from the Caribbean and Africa have a higher incidence, earlier onset aggressive breast cancers that are treatment refractory causing premature deaths than other ancestral groups. Combining ultra-highplexed protein spatial phenotyping with machine learning, they identified novel immune cell populations in unique stromal cell neighbourhoods deciphering more aggressive tumours by their stemness. Alex Swarbrick (Garvan Institute, Australia) utilised single cell and spatial transcriptomics to demonstrate the distinct programs and spatial distribution of specific cancer-associated fibroblast (CAF) subtypes. He discussed analyses of their ligand-receptor interactions and prediction of TFs that mediate stromal transitions to derive more effective therapeutic strategies. Brooke Pereira (Garvan Institute, Australia) used advanced proteomics and intravital microscopy to identify matrix signatures from highly metastatic, therapy resistant pancreatic tumours in mice and pinpoint drivers of fibrosis.

## Novel therapeutic and therapy resistance

Raelene Enderersby (Telethon Kids Institute, Australia) described advances in preclinical models for paediatric medulloblastoma developing a new patient derived orthotopic xenograft (PDOX) model in juvenile mice. The PDOX model revealed differences in the juvenile and adult brain immune tumour microenvironment providing a more relevant model to identify optimal immunotherapeutic approaches for this paediatric disease.

## Systems biology and cancer

Enrico Petretto (Duke-National University of Singapore) described a systems genetics approach to identify a pro-fibrotic gene network in multiorgan fibrosis and showed that the E3 ubiquitin ligase WWP2 N-terminal isoform is a master regulator of this network and tissue fibrosis. Transgenic mice lacking the N-terminus of WWP2 exhibited reduced tissue fibrosis in heart, lung and kidney. Alistair Forrest (Harry Perkins Institute, Australia) presented a spatial transcriptomics approach to define the clonal architecture of ovarian cancer and their microenvironments. Intratumor differences in copy number alterations and differential gene expression identified rewiring of cell-to-cell communication networks.

## Cancer metabolism

Kristin Brown (Peter MacCallum Cancer Centre, Australia) showed that amino acid withdrawal cause downregulation of major histocompatibility complex I (MHC-I) expression by induction of the ribotoxic stress response, and discussed the therapeutic potential of targeting amino acid metabolism to induce MHC-I expression for increased presentation to T-cells, thereby reducing immunotherapy resistance.

## Cancer biology to therapy

Heidi Neubauer (University of Veterinary Medicine, Vienna) described the first preclinical murine model for the rare and aggressive haematological malignancy Hepatosplenic T-cell lymphoma (HSTL). This model recapitulated human HSTL features including clinically relevant STAT5 gain-of-function mutations. The opportunity to pharmacologically target the pseudokinase, mixed lineage kinase domain-like (MLKL), to control the necroptosis cell death pathway was discussed by James Murphy (Walter and Eliza Hall Institute of Medical Research, Australia). He highlighted the role of MLKL in inflammatory bowel disease, which is associated with increased risk of bowel cancer.

## Tumour microenvironment

Valerie Weaver (University of California San Francisco, USA) discussed how ECM stiffness remodelling and increased mechanosignalling drive epithelial-to-mesenchymal transition and tumour metastasis. In particular, the metabolic reprogramming of macrophages into immunosuppressive phenotypes induced by stiffened ECM was highlighted. Maté Biro (University of New South Wales, Australia) described the development of advanced live cell microscopy for high spatiotemporal resolution imaging and analysis of mechanical forces exerted by cytotoxic T lymphocytes as they migrate in the three-dimensional collagen matrix to interact with and attack cancer cells. Thomas Cox (Garvan Institute, Australia) discussed the discovery of small molecules targeting lysyl oxidase (LOX), which is essential for collagen deposition, ECM integrity and tumour desmoplasia. This led to the development of PXS-5505, a first in-class selective pan-LOX inhibitor which inhibits ECM stiffness, CAF activation and tumour metastasis.

### Clifford prize presentation

The Clifford Prize for international excellence in cancer research was awarded to Ravindra Majeti for his seminal longstanding work in the identification and functional validation of AML leukaemic stem cell (LSC) populations and hierarchy (Fig. 1B). This lecture described Ravindra’s post-doctoral training in Irving Weissman’s laboratory where they discovered that CD47 expression on LSCs functions as a “do not eat me” signal for macrophage phagocytosis. Subsequently, Ravindra developed a humanised anti-CD47 antibody and initiated first-in-human clinical trials.

The 10^th^ Barossa Meeting brought together leading international scientist to discuss the genesis of cancer development and progression to uncover unique fundamental discoveries that will lead to potential therapeutic development. We hope to see you at the 11^th^ Barossa meeting in November 2025.

